# Developing high-transmittance heterojunction diodes based on NiO/TZO bilayer thin films

**DOI:** 10.1186/1556-276X-8-206

**Published:** 2013-05-01

**Authors:** Chia-Cheng Huang, Fang-Hsing Wang, Chia-Ching Wu, Hong-Hsin Huang, Cheng-Fu Yang

**Affiliations:** 1 Department of Electrical Engineering, National Chung Hsing University, Taichung, 402, Taiwan; 2 Department of Electronic Engineering, Kao Yuan University, Kaohsiung, 821, Taiwan; 3 Department of Electrical Engineering, Cheng-Shiu University, Kaohsiung, 833, Taiwan; 4 Department of Chemical and Materials Engineering, National University of Kaohsiung, Kaohsiung, 811, Taiwan

**Keywords:** Titanium-doped zinc oxide, Nickel oxide, Heterojunction diode, Space-charge limited current

## Abstract

In this study, radio frequency magnetron sputtering was used to deposit nickel oxide thin films (NiO, deposition power of 100 W) and titanium-doped zinc oxide thin films (TZO, varying deposition powers) on glass substrates to form p(NiO)-n(TZO) heterojunction diodes with high transmittance. The structural, optical, and electrical properties of the TZO and NiO thin films and NiO/TZO heterojunction devices were investigated with scanning electron microscopy, X-ray diffraction (XRD) patterns, UV-visible spectroscopy, Hall effect analysis, and current-voltage (*I*-*V*) analysis. XRD analysis showed that only the (111) diffraction peak of NiO and the (002) and (004) diffraction peaks of TZO were observable in the NiO/TZO heterojunction devices, indicating that the TZO thin films showed a good *c*-axis orientation perpendicular to the glass substrates. When the sputtering deposition power for the TZO thin films was 100, 125, and 150 W, the *I*-*V* characteristics confirmed that a p-n junction characteristic was successfully formed in the NiO/TZO heterojunction devices. We show that the NiO/TZO heterojunction diode was dominated by the space-charge limited current theory.

## Background

Transparent electronics is an advanced technology concerning the creation of invisible electronic devices. To realize transparent electronic and optoelectronic devices, transparent conducting oxides (TCOs) have been widely utilized [1-3]. Zinc oxide (ZnO) is an n-type semiconductor with a large binding energy of 60 meV and a wide bandgap of 3.3 eV. Doped ZnO thin films are promising alternatives to replace indium-tin oxide (ITO) thin films as TCOs due to the former's stable electrical and optical properties. The low resistivity of ZnO-based thin films arises from the presence of oxygen vacancies and zinc interstitials [4]. Aluminum (Al) [5], gallium (Ga) [6], and indium (In) [7,8] have been widely studied as dopants to enhance the n-type conductivity of ZnO-based thin films. ZnO-based TCO materials have numerous potential applications in electronic and optoelectronic devices, such as solar cells [9], light-emitting diodes [10], blue laser diodes [11], and flat-panel displays [12]. Trivalent cation-doped ZnO thin films present good electrical conductivity and transparency over the visible spectrum. In the past, Chung et al. investigated the properties of Ti-doped ZnO thin films with different TiO_2_ concentrations and reported that the lowest resistivity of TZO thin films was achieved when the Ti concentration was 1.34 mol% [13]. Lin et al. studied the effect of substrate temperature on the properties of TZO thin films by simultaneous radio frequency (RF) and DC magnetron sputtering [14]. Wang et al. examined the effects of substrate temperature and hydrogen plasma treatment on the characteristics of TZO thin films [15].

Nickel oxide (NiO) is a p-type semiconductor TCO material with a wide range of applications: it has been used in transparent conductive films [16] and electrochromic devices [17] and as a functional layer material in chemical sensors [18]. NiO has a wide bandgap of 3.6 to 4.0 eV at room temperature; hence, a NiO thin film is also transparent in the range of visible light [19]. According to the literature, TZO and NiO thin films can be prepared by sputtering [16,20], chemical vapor deposition [21,22], and the sol-gel process [23,24]. Among these methods, sputtering is the most widely used. In this paper, the fabrication and characterization of an optically transparent p-n heterojunction diode by deposition of NiO thin films on TZO thin films are presented, with an emphasis on device performance, including transparent and current-voltage characteristics. In addition, the structural, optical, and electrical properties of the NiO/TZO heterojunction diodes were characterized by scanning electron microscopy (SEM), X-ray diffraction (XRD) patterns, UV-visible spectroscopy, and Hall effect measurement.

## Methods

**Figure 1:**
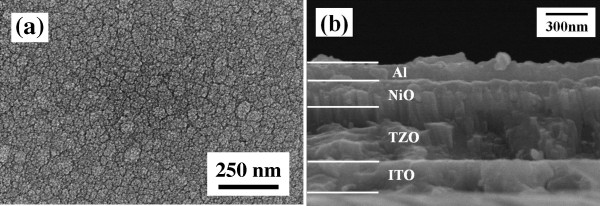
**Images of a NiO/125 W-deposited TZO heterojunction diode. (a)** Surface and **(b)** cross-sectional SEM images.

The raw materials (ZnO and TiO_2_) were weighed according to the composition formula ZnO = 98.5 mol% and TiO_2_ = 1.5 mol% (TZO) and ball-milled with deionized water for 1 h. After being dried and ground, the powder was uniaxially pressed into a 2-in. plate in a steel die, and sintering was carried out at 1,350°C in air for 2 h. High-purity NiO powder was sintered at 1,500°C to prepare the ceramic target. TZO thin films were deposited on 25 mm × 25 mm × 1 mm ITO glass (7 Ω/per square area) substrates; then, NiO thin films were deposited on the TZO using a Syskey 13.56 MHz RF magnetron sputtering system (Syskey Technology Ltd., Hsinch County, Taiwan). The deposition power was 100 W for the NiO thin films and was changed from 75 to 150 W for the TZO thin films. The working distance between the substrate and target was fixed at 5 cm. The base pressure was 5 × 10^−6^ Torr, and the working pressure was maintained at 5 × 10^−3^ Torr. After the TZO and NiO thin films were deposited, a circle Al electrode of 1 mm in diameter was deposited on the NiO thin films (as shown in Figure 1b). The crystalline structures of the TZO and NiO thin films were determined with an X-ray diffractometer using CuKα radiation (*K* = 1.5418 Å). The deposition times of the NiO and TZO thin films were 10 and 20 min, respectively. The film thicknesses were measured using a Nano-view SEMF-10 ellipsometer (Nano-View Co., Ltd., Ansan, South Korea) and confirmed by a field emission scanning electron microscope. The mobility, carrier concentration, and resistivity were obtained from Hall effect measurements using the Van der Pauw method (HMS-3000, Ecopia, Anyang-si, South Korea). Optical transmittance was measured using a UV/vis/IR spectrophotometer (V-570, JASCO Inc., Easton, MD, USA) in the 250- to 2,500-nm wavelength range. The current-voltage (*I*-*V*) characteristics of the NiO/TZO heterojunction diodes were measured by an HP4156 semiconductor parameter analyzer (Hewlett-Packard, Palo Alto, CA). The measurements were performed by changing the bias voltage from +10 to −10 V.

## Results and discussion

**Figure 2:**
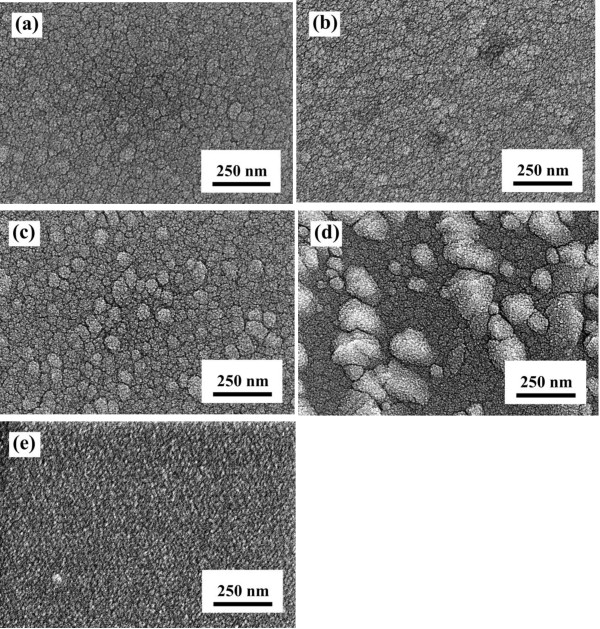
**Surface SEM images of TZO and NiO thin films as a function of deposition power.** TZO thin films were deposited at **(a)** 75 W, **(b)** 100 W, **(c)** 125 W, and **(d)** 150 W; **(e)** the NiO thin film deposited at 100 W.

Surface SEM images of the TZO and NiO thin films are shown in Figure 2. In Figure 2a, b, when the deposition power was 75 and 100 W, respectively, the surface morphologies of the TZO thin films are smooth and not compact. Particle aggregation in the TZO thin films appeared to increase as the deposition power increased from 100 to 150 W, as shown in Figure 2b, c, d. This particle aggregation can be attributed to a high deposition rate due to the high-energy plasma when the deposition power was 125 and 150 W. However, as the deposition power was increased to 150 W, the roughness of the TZO thin films increased because of the large aggregations of particles. In Figure 2e, by contrast, the 100 W-deposited NiO thin film has a smooth and uniform surface.

**Figure 3:**
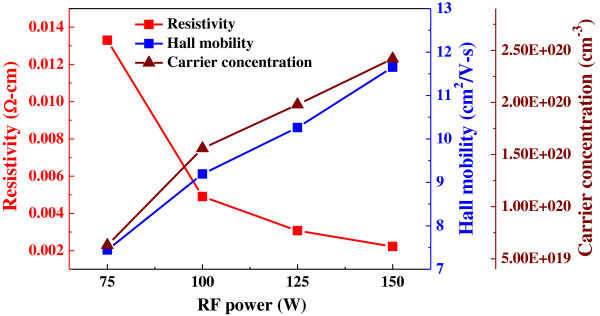
Resistivity, hall mobility, and carrier concentration of TZO thin films as a function of deposition power.

NiO deposited at 100 W had a hall mobility of 6.19 cm^2^/V s, carrier concentration of 4.38 × 10^20^ cm^−3^, and resistivity of 2.2 × 10^–3^ Ω cm (not shown here). Figure 3 shows the resistivity, hall mobility, and carrier concentration of the TZO thin films as a function of deposition power. Electrons generated from oxygen vacancies and Zn interstitial atoms resulting from the dopant primarily determine the conduction properties of TZO thin films. Therefore, the films' electrical conductivity will exhibit large variations when different deposition powers are used. As the deposition power was increased from 75 to 150 W, the hall mobility increased from 7.45 to 11.69 cm^2^/V s, and the carrier concentration increased from 2.75 × 10^19^ to 4.38 × 10^20^ cm^−3^. The higher hall mobility and carrier concentration are due to the higher deposition power; as it increases from 75 to 150 W, the kinetic energy of the deposited molecules increases, so more molecules can diffuse and deposit onto the surfaces of the glass substrates. Consequently, the TZO thin films will have better crystal quality and larger particle aggregations. Therefore, a reduced grain boundary barrier is obtained, leading to an increase in carrier mobility. The resistivity of TCO thin films is proportional to the reciprocal of the product of carrier concentration (*N*) and hall mobility (*μ*):

(1) ρ=1/Neμ

which is a combined result of both the mobility and the carrier concentration. The resistivity of TZO thin films linearly decreased from 1.3 × 10^−2^ to 2.2 × 10^−3^ Ω cm when the deposition power was increased from 75 to 150 W.

**Figure 4:**
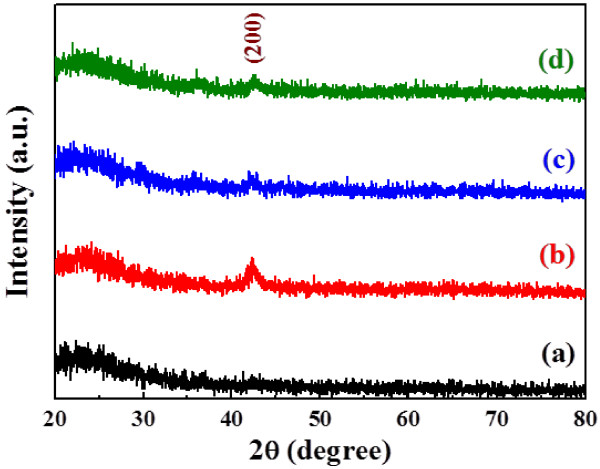
**XRD patterns of NiO thin films as a function of deposition power. (a)** 75 W, **(b)** 100 W, **(c)** 125 W, and **(d)** 150 W.

**Figure 5:**
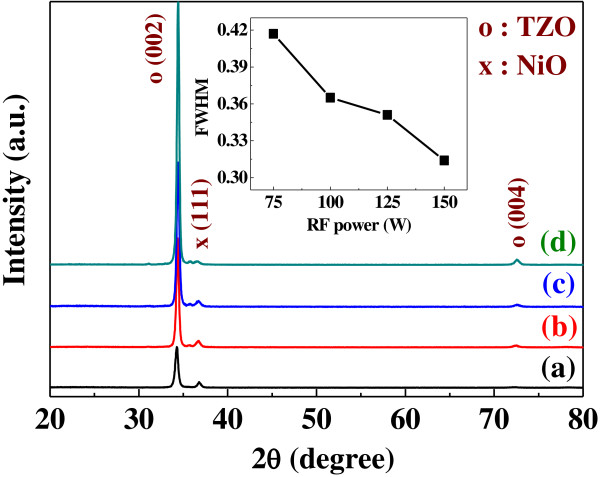
**XRD patterns of NiO/TZO heterojunction diodes as a function of deposition power of TZO thin films. (a)** 75 W, **(b)** 100 W, **(c)** 125 W, and **(d)** 150 W.

The surface SEM image of a heterojunction diode formed by using a 100 W-deposited NiO thin film on 125 W-deposited TZO thin film is shown in Figure 1a; the morphology was similar to that of the 125 W-deposited TZO thin film. Also, the surface morphologies of the 100 W-deposited NiO thin film on the 100 W-deposited and 150 W-deposited TZO thin films were similar to the results of the 100 W-deposited and 150 W-deposited TZO thin films (Figure 2b, d, not shown here). Those results demonstrate that the surface morphologies of the TZO thin films deposited at different powers will dominate the surface crystalline structure of NiO thin films. The XRD patterns compared in Figure 4 (for NiO thin films) and Figure 5 (for NiO/TZO thin films) will also demonstrate that the TZO thin films can dominate the crystalline structure of NiO thin films. The uniformity and roughness of the 100 W-deposited NiO/125 W-deposited TZO heterojunction diode were better than those of the NiO/TZO heterojunction diodes with TZO thin films deposited at other powers (not shown here). Figure 1b shows the cross-section SEM image of the 100 W-deposited NiO/125 W-deposited TZO heterojunction diode; the Al electrode and the ITO substrate electrode are also observed in Figure 1b. Cross-sectional observations of all the NiO/TZO heterojunction diodes showed that NiO thin films deposited on different TZO thin films had the same thickness of about 180 nm, which was achieved by controlling the deposition time. However, although the TZO thin films were deposited in the same amount of time, they had thicknesses of about 315, 350, 380, and 450 nm as the deposition power was changed from 75 W (not shown here) to 100 W (not shown here), 125 W, and 150 W (not shown here), respectively.

Figure 4 shows the XRD patterns of the NiO thin films deposited as a function of deposition power. No matter what deposition power was used, the only (200) diffraction peak was observed in the NiO thin films, and the 100 W-deposited NiO thin films had the optimal crystallization. XRD patterns of the NiO/TZO heterojunction diodes for TZO thin films deposited at different deposition powers are shown in Figure 5. All patterns exhibited the (002) and (004) diffraction peaks of the ZnO (TZO) crystallization preferential orientation along the *c*-axis at diffraction angles (2*θ*) near 34.28° and 72.58°, with a hexagonal structure; no peak characteristic of TiO_2_ was found. The diffraction peak revealed that a 2*θ* value of 36.74° corresponded to the (111) plane of the NiO thin film with a cubic structure, which was different from the result in Figure 4. The result in Figure 5 is an important proof that as the NiO thin films is deposited on the TZO thin films with the (002) and (004) diffraction peaks, the crystalline structure of the NiO thin films will be controlled by TZO thin films. For that, the main diffraction peak is changed from the (200) plane to the (111) plane, and then the TZO thin films will dominate the crystalline structure (Figure 1a). Figure 5 also shows that both the diffraction intensity ratio of 2*θ*_TZO(002)_/2*θ*_NiO(111)_ and the diffraction intensity of the TZO thin films increased with increasing deposition power. In addition, as the deposition power increased from 75 to 150 W, the full width at half maximum (FWHM) values decreased from 0.417 to 0.314, as shown in the inset of Figure 5. Those results reveal that the crystallization of TZO thin films is enhanced at higher deposition powers. This finding proves that the resistivity of TZO thin films closely depends on variations in deposition power (see Figure 3) because the crystallization of TZO thin films increases as the FWHM value decreases [14].

**Figure 6:**
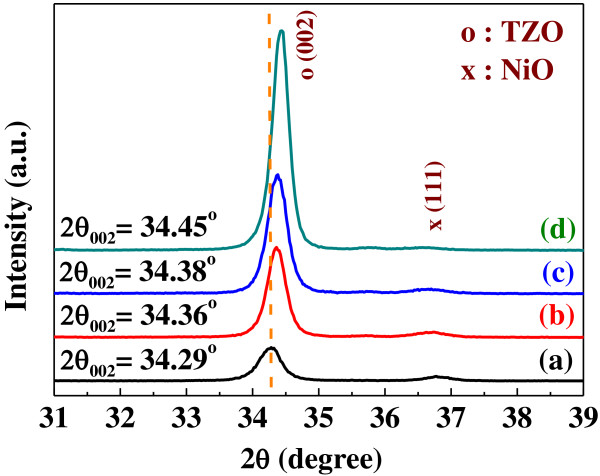
**GIAXRD patterns of NiO/TZO heterojunction diodes as a function of deposition power of TZO thin films. (a)** 75 W, **(b)** 100 W, **(c)** 125 W, and **(d)** 150 W.

The grazing incidence angle X-ray diffraction (GIAXRD) patterns of NiO/TZO heterojunction diodes in the 2*θ* range of 31° to 39° are shown in Figure 6. The diffraction spectra show that the 2*θ* value of the (002) peak shifted from 34.29° to 34.45° as the deposition power of the TZO thin films increased from 75 to 150 W. This may be attributed to the fact that as higher deposition power is used, higher crystallization of the TZO thin films is obtained, and the effect for Ti atoms to substitute the sites of Zn atoms is more apparently revealed. Since the ionic radius of Ti^4+^ (68 pm) is smaller than that of Zn^2+^ (74 pm), the 2*θ* value of the (002) peak is expected to shift upwards.

**Figure 7:**
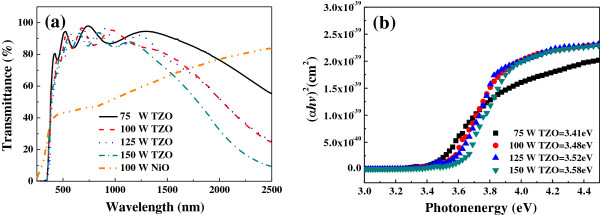
**TZO thin films. (a)** Transmittance and **(b)***αhυ*^2^ vs. *E*_*g*_ plots of the TZO thin films as a function of deposition power.

The optical transmittance spectra of TZO and NiO thin films in the wavelength range from 250 to 2,500 nm are shown in Figure 7a. The average transmittance rate of TZO thin films is about 90% in the 400- to 1,200-nm range, even when different deposition powers are used, and the average transparency of the NiO thin film is about 45% in the 400- to 700-nm range. In the ultraviolet range, all of the TZO thin films showed a sharp absorption edge and exhibited a blueshift phenomenon with increasing deposition power, as shown in the results in Figure 7b. This blueshift can be explained by the Burstein-Moss shift, a shift of the Fermi level into the conduction band, the energy of which enhances the optical bandgap [25,26]:

(2) $$\Delta E_{g}^{\mathrm{BM}}=\frac{\hbar^{2}k_{F}^{2}}{2}\left(\frac{1}{m_{e}}+\frac{1}{m_{h}}\right)=\frac{\hbar^{2}k_{F}^{2}}{2m_{\mathrm{vc}}^{*}},$$

where *k*_F_ stands for the Fermi wave vector and is given by *k*_F_ = (3π^2^*n*_*e*_)^1/3^; *m*_e_ is the effective mass of electrons in the conduction band, and *m*_h_ is the effective mass of holes in the valence band, which can be simplified as the reduced effective mass $m_{\mathrm{vc}}^{*}$. $\Delta E_{g}^{\mathrm{BM}}$ can be rewritten by inducing *k*_F_ for the carrier concentration *n*_*e*_:

(3) $$\Delta E_{g}^{\mathrm{BM}}=\frac{\hbar^{2}}{2m_{\mathrm{vc}}^{*}}{\left(3\pi^{2}n_{e}\right)}^{2/3}.$$

Equation 3 shows that the Burstein-Moss shift of the absorption edge to the shorter wavelength region is due to the increase in carrier concentration (*n*_*e*_), as demonstrated in Figure 3.

**Figure 8:**
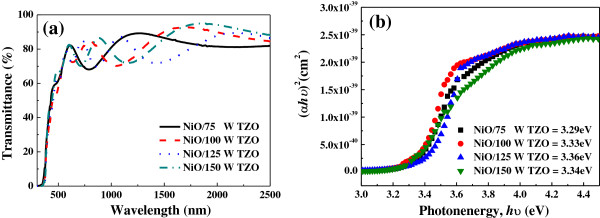
**NiO/TZO heterojunction diodes. (a)** Transmittance and **(b)***αhυ*^2^ vs. *E*_*g*_ plots of NiO/TZO heterojunction diodes.

Figure 8 shows the transmittance spectra of the NiO/TZO heterojunction diodes as a function of the TZO thin films' deposition power. The optical transmittance at 400 to 700 nm is more than 80% for all of the NiO/TZO heterojunction diodes, regardless of the deposition power of the TZO thin films. Compared with Figure 7a, the results in Figure 8a show that the transmittance rate of the NiO/TZO heterojunction bilayer thin films were higher than that of the NiO thin film. The results in Figures 2 and 3 prove that the surface morphologies and crystalline structures of the bilayer NiO/TZO thin films are dominated by the TZO thin films. For that, the transmittance rate of the NiO/TZO heterojunction bilayer thin films is also dominated by the TZO thin films and will be higher than that of the NiO thin film. All of the NiO/TZO heterojunction diodes showed a sharp absorption edge, but they did not exhibit the blueshift phenomenon as the deposition power of the TZO thin films increased. Compared with other research, the NiO/TZO heterojunction diodes obtained in this study have the highest transmittance, even higher than that of deposited NiO thin films. The corresponding optical bandgap (*E*_*g*_) was determined by applying the Tauc model and the Davis and Mott model [27] using Equation 4:

(4) $${\left(\mathrm{\alpha h\upsilon}\right)}^{2}=c\left(\mathrm{h\upsilon}-E_{g}\right)$$

where *α* is the optical absorption coefficient, *c* is the constant for direct transition, *h* is Planck's constant, and *υ* is the frequency of the incident photon. Figure 8b shows (*αhυ*)^2^ vs. *hυ* for the NiO/TZO heterojunction diodes. Their *E*_*g*_ values increased when the deposition power of the TZO thin films increased from 75 to 125 W. The variations in *E*_*g*_ values roughly agree with those of the carrier concentrations shown in Figure 3.

**Figure 9:**
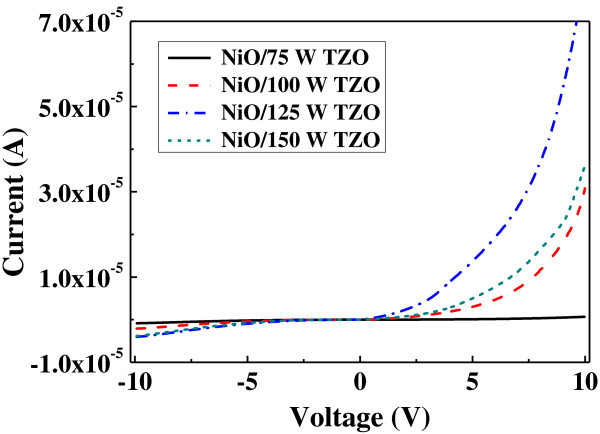
***I*-*V *characteristics of the NiO/TZO heterojunction diodes.**

Figure 9 shows the *I*-*V* characteristics of the NiO/TZO heterojunction diodes. The nonlinear and rectifying *I*-*V* characteristics confirmed that a p-n junction diode was successfully formed in the NiO/TZO heterojunction structure. In the forward bias condition, the turn-on voltages of the NiO/TZO heterojunction diodes were about 2.57, 1.83, and 2.05 V as the deposition powers of the TZO thin films were 100, 125, and 150 W, respectively. The turn-on voltage of the NiO/TZO heterojunction diodes decreased as the deposition power increased from 75 to 125 W; then, it increased with a 150-W deposition power. As the deposition power increased from 75 to 125 W, the resistivity linearly decreased (Figure 3), causing the decrease in turn-on voltage. However, even though TZO thin films deposited at 150 W have lower resistivity, the increase in turn-on voltage is due to the greater roughness of the TZO thin film (Figure 2d) and the defects that exist between the p-n heterojunction interfaces of the NiO and TZO thin films. In addition, the forward currents of the NiO/TZO heterojunction diodes abruptly increase when the turn-on voltages are over 2.57 V (deposition power 100 W), 1.83 V (125 W), and 2.05 V (150 W), which demonstrates that the *I*-*V* curves are a characteristic of a typical p-n junction diode. For TZO thin films deposited at 75 W, the symmetrical *I*-*V* curve of the NiO/TZO heterojunction device is not a typical characteristic of a p-n junction diode.

**Figure 10:**
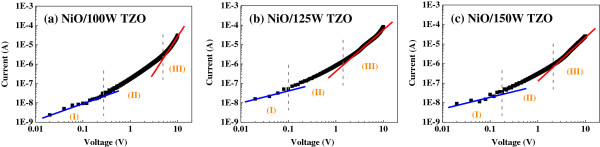
**Log(*I*)-log(*V*) characteristics of NiO/TZO heterojunction diodes as function of deposition power of TZO thin films. (a)** 100 W-deposited TZO, **(b)** 125 W-deposited TZO, and **(c)** 150 W-deposited TZO.

The log (*I*)-log (*V*) plots in Figure 10 clearly show the power law behavior of current and voltage, which can be used to find the behavior of the charge transport in Figure 9. Figure 10 proves that the space-charge limited current (SCLC) theorem dominates the mechanism of the *I*-*V* curves in the structure of the NiO/TZO heterojunction diodes [23,24]. Because the NiO/75 W-deposited TZO heterojunction device had a symmetrical *I*-*V* curve, as forward and reverse voltages were used and the current was small, as +10 and −10 V were used as bias, the SCLC theorem was not used to explain its mechanism. A low forward voltage for *V* < 0.4 V (0.26, 0.097, and 0.17 V for deposition powers of 100, 125, and 150 W, respectively) indicates a transport mechanism obeying the Ohmic law at region (I). The value of the forward voltage decreases as the deposition power of the TZO thin films increases from 75 to 125 W, but the value of the forward voltage increases when the deposition power of the TZO thin films is 150 W.

From the above results, we know that the variations in forward voltage are similar to the turn-on voltages of the NiO/TZO heterojunction diodes. In the high forward voltage region (III), the voltages are large 4.7, 1.3, and 2.1 V for TZO thin film deposition powers of 100, 125, and 150 W, respectively, and those results are dominated by the SCLC mechanism. The transition region (II), between regions (I) and (III), often appears in SCLC-dominated *I*-*V* characteristics when traps are used. The presence of trap bands with different energies is responsible for different slopes in the different regions of the *I*-*V* characteristics. The results obtained in this study indicate that the charge transport mechanism of the investigated diodes can be influenced by the SCLC.

## Conclusions

In this study, the resistivity of TZO thin films linearly decreased from 1.3 × 10^−2^ to 2.2 × 10^−3^ Ω cm, and the average transparency of TZO thin films was about 90% in the wavelength range from 400 to 1,200 nm as the deposition power increased from 75 to 150 W. Transparent p-n heterojunction diodes were successfully fabricated using NiO and TZO thin films. These NiO/TZO heterojunction diodes had an average transparency of over 82% in the visible region. For TZO thin films deposited at 75 W, the symmetrical *I*-*V* curve of the NiO/TZO heterojunction diodes was not a typical characteristic of a p-n junction diode. The forward currents of the NiO/TZO heterojunction diodes abruptly increased when the turn-on voltages were over 2.57 V (deposition power 100 W), 1.83 V (125 W), and 2.05 V (150 W), demonstrating that these *I*-*V* curves are a characteristic of a typical p-n junction diode. The log scale of these *I*-*V* curves indicated that the SCLC dominates the current transport.

## Competing interests

The authors declare that they have no competing interests.

## Authors’ contributions

C-C H carried out the experimental procedures, including the depositions of NiO and TZO thin films and measurements of SEM and X-ray patterns. F-H W gave the suggestion for the paper organization and English grammar correction. C-F Y participated in the design of the study, performed the statistical analysis, and organized the paper. C-C W and H-H H participated in the measurement and prediction of the *I*-*V* curve of NiO/TZO heterojunction diodes using the space-charge limited current (SCLC) theorem. All authors read and approved the final manuscript.
